# Nystagmus Estimation for Dizziness Diagnosis by Pupil Detection and Tracking Using Mexican-Hat-Type Ellipse Pattern Matching

**DOI:** 10.3390/healthcare9070885

**Published:** 2021-07-13

**Authors:** Yoanda Alim Syahbana, Yokota Yasunari, Morita Hiroyuki, Aoki Mitsuhiro, Suzuki Kanade, Matsubara Yoshitaka

**Affiliations:** 1 Graduate School of Engineering, Gifu University, Yanagido 1-1, Gifu 501-1193, Japan; 2 Computer Engineering, Information Technology Department, Politeknik Caltex Riau, Umban Sari No. 1, Riau 25265, Indonesia; 3 Department of Electrical, Electronics and Computer Engineering, Faculty of Engineering, Gifu University, Yanagido 1-1, Gifu 501-1193, Japan; ykt@gifu-u.ac.jp; 4 Department of General Medicine and General Internal Medicine, Gifu University Hospital, Gifu University, Yanagido 1-1, Gifu 501-1194, Japan; hmorita@gifu-u.ac.jp; 5 Department of Otolaryngology, Graduate School of Medicine, Gifu University, Yanagido 1-1, Gifu 501-1194, Japan; aoki@gifu-u.ac.jp; 6 Center for Healthcare Information Technology, Tokai National Higher Education and Research System, Furo-cho, Chikusa-ku, Nagoya 464-8601, Japan; 7 Medical IT Support Department, HRS Co., Ltd., Room B, 10th Floor, Itochu Marunouchi Building, 1-5-28 Marunouchi, Naka-ku, Nagoya 460-0002, Japan; kan_suzuki@hrs-grp.co.jp (S.K.); y_matsubara@hrs-grp.co.jp (M.Y.); 8 Department of Otolaryngology-Head and Neck Surgery, School of Medicine, Gifu University, Yanagido 1-1, Gifu 501-1194, Japan

**Keywords:** video oculography, nystagmus analysis, pupil detection and tracking, pattern matching, Mexican hat-type ellipse pattern

## Abstract

The detection of nystagmus using video oculography experiences accuracy problems when patients who complain of dizziness have difficulty in fully opening their eyes. Pupil detection and tracking in this condition affect the accuracy of the nystagmus waveform. In this research, we design a pupil detection method using a pattern matching approach that approximates the pupil using a Mexican hat-type ellipse pattern, in order to deal with the aforementioned problem. We evaluate the performance of the proposed method, in comparison with that of a conventional Hough transform method, for eye movement videos retrieved from Gifu University Hospital. The performance results show that the proposed method can detect and track the pupil position, even when only 20% of the pupil is visible. In comparison, the conventional Hough transform only indicates good performance when 90% of the pupil is visible. We also evaluate the proposed method using the Labelled Pupil in the Wild (LPW) data set. The results show that the proposed method has an accuracy of 1.47, as evaluated using the Mean Square Error (MSE), which is much lower than that of the conventional Hough transform method, with an MSE of 9.53. We conduct expert validation by consulting three medical specialists regarding the nystagmus waveform. The medical specialists agreed that the waveform can be evaluated clinically, without contradicting their diagnoses.

## 1. Introduction

Dizziness is a common symptom presented by patients in a health examination [1]. Dizziness represents an unsteady sensation accompanied by a feeling of movement within the head [2]. Based on [3], the four categories of dizziness are lightheadedness, presyncope, disequilibrium, and vertigo. Among these categories, vertigo is the most common cause of dizziness, which is related to neurological conditions [4]. Two categories of vertigo are central vertigo, related to disease/injury in the brain, and peripheral vertigo, related to a vestibular disorder. In terms of signs and symptoms, vertigo has many potential causes, and the symptoms can be vague, non-specific, and inconsistent [5]. As dizziness due to vertigo is a subjective symptom, the symptom threshold depends on the patient’s sensitivity [6]. Correlating dizziness and its cause has become a significant challenge for medical specialists, as the cause of dizziness determines the treatment offered for dizziness [3].

Based on [7,8,9,10], medical specialists can use nystagmus symptoms as a crucial element in identifying the cause of dizziness. Different types of nystagmus can be categorized by analyzing the fast and slow phase or the alternating slow phase of eye movement. Existing studies of nystagmus have provided a review of the critical clinical literature, in order to support state-of-the-art differential diagnosis [11], and have discussed the clinical features of nystagmus and its relation to ocular motility disorder [12,13,14]. Existing studies have also focused on the treatment and therapy process [15,16], and case-by-case of nystagmus in specific subject categories [17,18] for different forms of nystagmus: vertical, positional, head-shaking and vibration-induced, and vestibular nystagmus.

Conventional observation, which is conducted visually by the medical specialist, can be subjectively biased. The visual examination also requires a medical specialist’s sufficient experience for accurate diagnosis. Furthermore, patients with dizziness may feel pain when attempting to consciously fully open their eyes, as such, their eye may remain only partially open. Therefore, an emphasis on nystagmus observation to support clinical decisions is essential, in order to enhance diagnostic reasoning by medical specialists [19]. A practical method is required to objectively measure eye movements and present the movement as a nystagmus waveform to the medical specialist.

An alternative method for eye movement measurement is video-oculography [20,21,22,23]. This method uses a camera to capture eye images, a computer to record the captured images, and software to detect and track eye movement. Due to advancements in camera technology and computer processing capability, video-oculography has become more popular and can serve as a more reliable method to measure eye movement [24]. In this research, we adopt the video-oculography method to obtain a nystagmus waveform for dizziness diagnosis. The waveform presents estimated eye movement, based on tracked pupil position from the patient’s eye. Initially, Frenzel goggles (equipped with an infrared camera and infrared illumination) capture eye images under night vision, with the light blocked by the goggles. Similarly, in this research, we use infrared light as a light source and an infrared camera to capture eye images. The light enters the pupil and diffuses inside the eyeball. Then, tissues and vitreous humor inside the eyeball absorb the diffused light. This process causes the pupil to become dark in the video frame. On the contrary, the iris and sclera will reflect the light, causing these areas to become bright [25]. Therefore, we tracked pupil position based on the high contrast between the iris and pupil, creating a boundary between the dark pupil area and the bright iris area.

In order to estimate patient pupil position accurately under the previously mentioned conditions, it is practical to model and use the pupil shape. In general, existing research uses a circle shape to approximate pupil shape [26], such as the circular Hough transform [23] method; however, the actual pupil shape is slightly flattened from a perfect circle and forms an ellipse. Approximating an ellipse shape with a model based on a circle causes deterioration of pupil estimation accuracy. In addition, a patient who complains of dizziness often has difficulty in opening their eyes fully. Therefore, this research proposes a pupil detection and tracking method using a Mexican-hat-type ellipse pattern, which can detect pupil position for a partially open pupil, as the main contribution of this paper.

This paper is organized into seven sections: Section 1 serves as an essential introduction to the research. Section 2 explains the working principle of the eye movement observation equipment used in this research. Section 3 describes the data sets that are used in this research. Section 4 presents the design of the proposed method, while Section 5 provides a discussion of the results using the proposed method. Section 6 deals with a performance evaluation of the proposed method. Section 7 concludes the paper, with a summary of the proposed method’s performance, the distinctive features of the proposed method based on medical specialists’ review, and the contributions of the research.

## 2. Working Principle of the Eye Movement Observation Equipment

**Figure 1:**
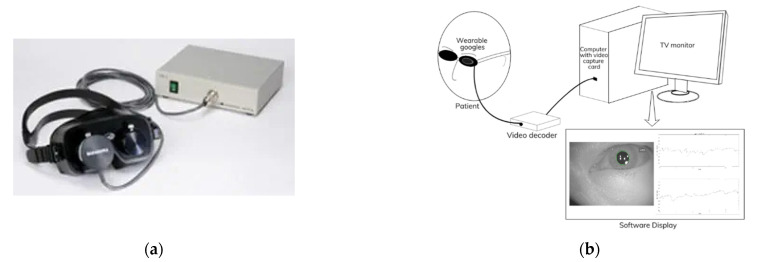
Eye movement observation equipment: (**a**) Infrared Eye Movement Imaging TV Device IEM-2 and video capture; and (**b**) system illustration.

Generally, eye movement observations associated with dizziness are conducted by preventing the visual fixation of patients’ eyes [3,12]. Therefore, the observation of nystagmus was conducted under night vision. We used the Infrared Eye Movement Imaging TV Device IEM-2 from Nagashima Medical Instrument Co. Ltd., shown in Figure 1a. The device includes wearable goggles with an infrared camera connected to a video decoder. The patient’s eyes were positioned inside the goggles to block the light from outside by a cover made of rubber. The goggles are also attached to an infrared light source that illuminates either the left or right eye of the patient; thus, the infrared camera can capture the eye. Then, the TV monitor presents the images captured by the camera, through a computer equipped with a video capture card. Figure 1b illustrates the system of eye movement observation equipment.

**Figure 2:**
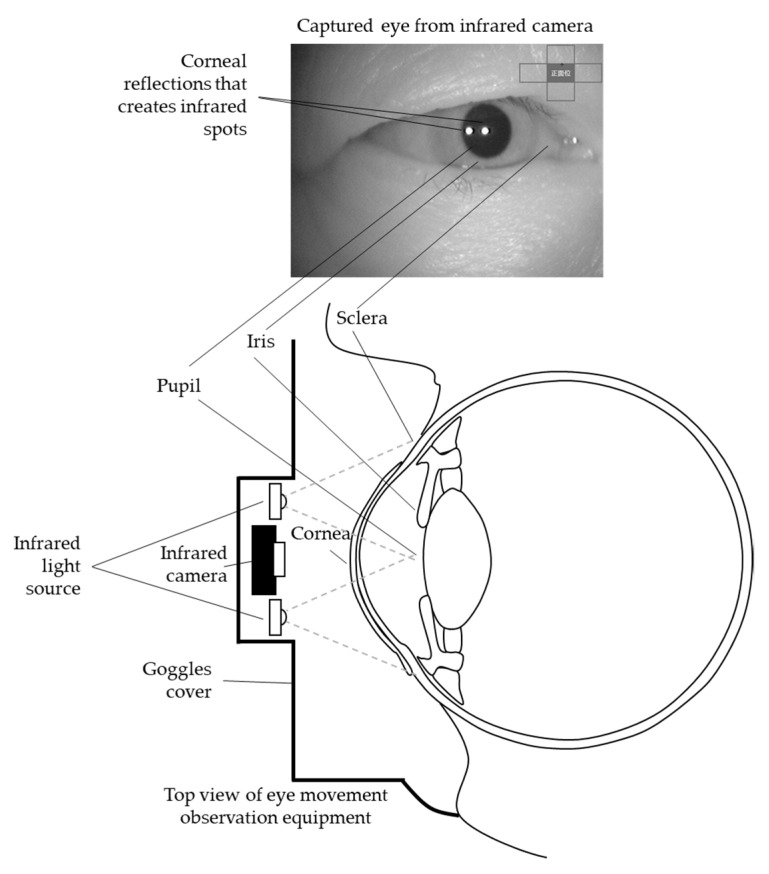
Illustration of eye movement observation equipment.

Eye movement observations using the abovementioned equipment were based on the dark-pupil technique. In this technique, the equipment illuminates the eye with an 887 nm near-infrared (NIR) light source and records the eye image with an infrared camera. The dark-pupil technique causes the pupil to become the darkest region in the image, as the eye is illuminated by an off-axis source. The light enters the pupil and diffuses inside of the eyeball. Then, the tissues and vitreous humor inside the eyeball absorb the diffused light. On the contrary, the iris, sclera, and eyelids reflect the light and appear bright in the eye image. This research uses an intensity gradient between the pupil and the iris to detect the pupil contour. The light also generates a corneal reflection of the light source, appearing as small and sharp glint dots. From now on, the dots are referred to as infrared spots. Figure 2 shows the working principle of the eye movement observation equipment used in this research.

## 3. Data Set Description

For this research, we used two data sets. The primary data set comprises eye movement videos obtained using the eye movement observation equipment explained in Section 2. The additional data set is the publicly available Labelled Pupil in the Wild (LPW) data set.

### 3.1. Eye Movement Video from Gifu University Hospital

The subjects in the eye movement videos were 22 males and 15 females aged from 28 to 81 years old. The subjects were diagnosed with semicircular canals or brain-related illnesses, such as Meniere’s disease, vestibular disorder, medulla oblongata bleeding, spinocerebellar degeneration, or multiple system atrophy. The eye movement videos of the subjects were retrieved from Gifu University Hospital. The videos show eye images with regular shape and good pupil transparency conditions. Table A1 in Appendix A summarizes the eye videos from these subjects.

A video frame from an eye movement video can be represented as I(x,y,t)∈{0,1,…,255}, $x\in \left\{1,2,\ldots ,N_{x}\right\}$, $y\in \left\{1,2,\ldots ,N_{y}\right\}$, and t∈{1,2,…,T}, where $N_{x}$ and $N_{y}$ are the width and height of the video frame, respectively, and T is the total number of video frames. The total video frames, T, was calculated as:

(1) T=Vduration∗Vfps,

where Vduration(s) is the duration of the video and Vfps (frame/s) is the video’s frame rate. In this research, $N_{x}$ = 640 pixels and $N_{y}$ = 480 pixels, except for videos 17, 18, 19, 24, and 27, which had $N_{x}$ = 720 pixels and $N_{y}$ = 480 pixels. In addition, video number 37 had $N_{x}$ = 320 and $N_{y}$ = 240 pixels. In this research, the video frame rate was Vfps = 30 frame/s. The total duration, Vduration, for each video used in this research is summarized in Table A1 in Appendix A.

### 3.2. Labelled Pupil in the Wild (LPW) Data Set

We evaluated the performance of the proposed method using the LPW data set [27]. This data set has been labeled with pupil center information as ground truth, for performance evaluation [28]. From the LPW data set, we selected I(x,y) for a total of 675 eye images, with $N_{x}$ = 384 pixels and $N_{y}$ = 288 pixels. The selection of I(x,y) was conducted based on pupil images of respondents that did not use glasses, eye contacts, or mascara in an indoor situation without strong reflection. We also selected I(x,y) captured from the front side, such that they resembled typical nystagmus observation images.

## 4. Proposed Method

**Figure 3:**
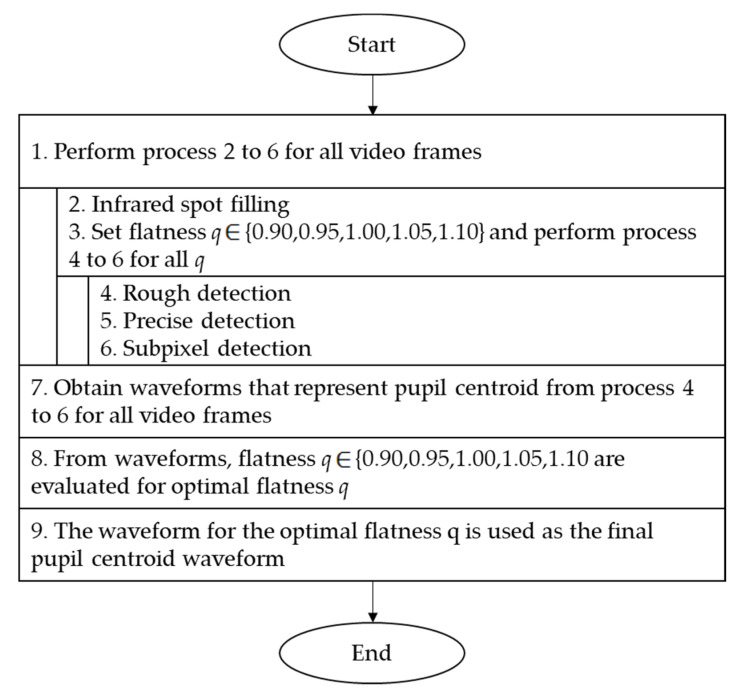
Design of proposed method.

Figure 3 shows the design of the proposed method, which is divided into nine processes. The details of each process are discussed in the following subsections.

### 4.1. Infrared Spot Filling

As previously explained in Section 2, infrared light was used as a light source. A transparent membrane can reflect infrared light on the surface of the cornea and create infrared spots. Processing is required to remove the reflected infrared spots in the video frame, as they produce strong edges and adversely affect the estimation of pupil position.

The brightness of this infrared spot was approximately represented by a high-intensity value (i.e., larger than 250). Therefore, the spot was detected by

(2) $$I_{spot}(x,y,t)=\{\begin{array}{c} 1,I(x,y,t)>250\\ 0,otherwise \end{array},$$

where $I_{spot}(x,y,t)$ is the detected reflection of the infrared spot. $I_{spot}(x,y,t)$ is a variable that takes a binary value, representing a pixel estimated to be an infrared spot with 1 and all others with 0. Around these spots, there exist regions with lower intensity values (i.e., I(x,y,t)<250), which are also part of the infrared spot reflection. Therefore, a dilation process was applied, in order to include the surrounding region. $I_{spot}(x,y,t)$ is dilated with a size of 7 × 7; thus, the surrounding region is also detected as an infrared spot. Then, a mean value of pixels in I(x,y,t) that surround over one pixel outside the infrared spot replaces the intensity value in the corresponding I(x,y,t) within the infrared spot region. After this step, I(x,y,t) is redefined as a video frame without an infrared spot.

Edge detection is performed on I(x,y,t) for each frame t. Several popular methods, including Sobel, Prewitt, Roberts, and Canny, were compared for the videos tabulated in Table A1, Appendix A. Comparing these methods, the Canny method had the best performance, and we decided to use the Canny edge detection method for our experiment. The edge detection result from the image I(x,y,t) is represented by $I_{edge}(x,y,t)$.

### 4.2. Estimation of Pupil Center Position and Radius

#### 4.2.1. Mexican Hat-Type Ellipse Pattern Matching

In order to detect the pupil as an ellipse, it is necessary to estimate the parameters of the ellipse, including the *x* coordinate, *y* coordinate, radius, flatness, and flattening direction of the center of the pupil. We confirmed that the pupil is flattened only in the vertical direction and stays equal in the horizontal direction, based on an examination of all eye movement videos. Therefore, the flat direction parameter of the ellipse was only focused on the vertical direction. The ellipse with a radius r centered at the coordinate $\left(x_{0},y_{0}\right)$ can be represented the set of points (x,y) satisfying the equation

(3) $${\left(\frac{x-x_{0}}{q}\right)}^{2}+{\left(y-y_{0}\right)}^{2}=r^{2},$$

where q is the flatness of the ellipse, which represents the ratio of the horizontal radius to the vertical radius of the ellipse. As an illustration, a perfect circle is obtained when q=1, a horizontally long ellipse is obtained when q>1, and a vertically long ellipse is obtained when q<1. A pattern matching process was performed on the edge image, $I_{edge}(x,y,t)$, using the generated ellipse pattern. The center coordinate $\left(x_{0},y_{0}\right)$, radius r, and flatness q were obtained by maximizing the evaluation function in the pattern matching process.

In order to define the evaluation function, the following two-dimensional function $f(x,y;x_{0},y_{0},r,q)$, as the ellipse pattern, was calculated using

(4) $$f(x,y;x_{0},y_{0},r,q)=(1-g(x,y;x_{0},y_{0},r,q))e^{-\frac{g(x,y;x_{0},y_{0},r,q)}{2}},$$

in which,

(5) $$g(x,y;x_{0},y_{0},r,q)={\left(\frac{\sqrt{{\left(\frac{x-x_{0}}{q}\right)}^{2}+{\left(y-y_{0}\right)}^{2}}-r}{\frac{r}{15}}\right)}^{2}.$$

**Figure 4:**
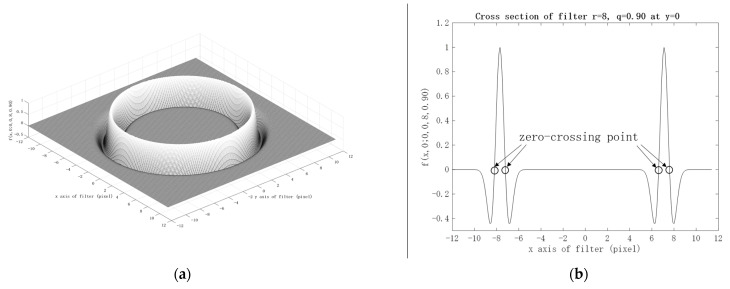
The example of the function $f(x,y;x_{0},y_{0},r,q)$, with $x_{0}=y_{0}=0$, r=8, and q=0.90: (**a**) Bird’s-eye view; and (**b**) cross-section at y = 0.

An example of the function $f(x,y;x_{0},y_{0},r,q)$, with $x_{0}=y_{0}=0$, r=8, and q=0.90, is shown in Figure 4. Figure 4a,b shows the bird’s-eye view and the cross-section at 𝑦 = 0 of the function, respectively. The r15 in Equation (5) represents the zero-crossing point into lateral suppression, marked by the black circles in Figure 4b. This optimal value was determined by some preliminary experiments on all eye movement videos. This Mexican hat-type ellipse pattern aims to concentrate the blurred edge of the pupil into a single sharp peak of the evaluation function. The Mexican hat-type shape will have maximum amplitude at a single peak and gradually suppresses insignificant edges. Therefore, the Mexican hat-type ellipse pattern can improve the accuracy of ellipse detection. A similar approach has also been studied, in order to improve the conventional Hough transform accuracy in detecting circle shapes, instead of the ellipse shape used in this research [29]. The result shows that the Mexican hat-type shape fitted the circle candidate and removed the fake circle associated with the conventional Hough transform. The term Mexican hat is used, due to its similarity to a Sombrero when plotted as a 2D image.

Initially, we investigated the ranges of radius and flatness for all eye movement videos for the subjects denoted in Table A1, Appendix A. Based on the investigation results, the radius r and flatness q were approximately varied, as 32≤r≤104 pixels and 0.90≤q≤1.10, respectively. Thus, the search range of pupil shape was defined, based on the radius r, as r∈{32,36,…,104} and, based on the flatness q, as q∈{0.90,0.95,1.00,1.05,1.10}.

The evaluation function, namely, the degree of similarity, was defined as:

(6) $$h(x_{0},y_{0},r,q;t)=\sum_{x=1}^{N_{x}}\sum_{y=1}^{N_{y}}f(x,y;x_{0},y_{0},r,q)I_{edge}(x,y,t),$$

for each frame t and flatness q. The calculation of Equation (6) is equivalent to a two-dimensional moving average filter for $I_{edge}(x,y,t)$ with filter coefficient $f(x,y;x_{0},y_{0},r,q)$. The pupil ellipse parameter center coordinate ($x_{0}$, $y_{0}$) and the radius r were estimated using the maximum value of the evaluation function $h(x_{0},y_{0},r,q;t)$. The parameters were written as $x_{0}(t)$, $y_{0}(t)$, and r(t), respectively, and $x_{0}$, $y_{0}$, and r were functions of the frame *t*.

#### 4.2.2. Three Steps Precision Improvement

In this research, approximating the pupil using an ellipse shape increased the number of parameters to be estimated and calculation cost, compared to the use of a circle shape. Therefore, we adopted a method for improving estimation accuracy consisting of three steps—rough, precise, and subpixel detection—to estimate the pupil center and radius mentioned in Section 4.2.1.

Initially, the rough detection estimation of the pupil center and radius from the entire image with an accuracy of 4 pixels was conducted. In order to detect a pupil with an accuracy of 4 pixels, the image I(x,y,t) (after infrared spot filling) was spatially down-sampled by 1/4. As a consequence, the search range r was also redefined as r∈{324,364,…,1044}. Then, $x_{0}(t)$, $y_{0}(t)$, and r(t) were estimated, using the method described in Section 4.2.1. Finally, these parameters were multiplied by four, in order to return them to the original scale.

Following this, the precise detection step used the estimated parameters $x_{0}(t)$, $y_{0}(t)$, and r(t) from the rough detection step, in order to crop the search range. The cropped image was defined by the ranges $x_{0}(t)-r(t)-w\leq x\leq x_{0}(t)+r(t)+w$ and $y_{0}(t)-r(t)-w\leq y\leq y_{0}(t)+r(t)+w$, where w is the width of the area included around the pupil. In this research, w=20 pixels were selected as the included area width. In the rough pupil detection step, the pupil center $\left(x_{0},y_{0}\right)$ and radius r were estimated with an accuracy of 4 pixels. Therefore, in the precise pupil detection step, the search ranges for the pupil center $\left(x_{0},y_{0}\right)$ and radius r were limited to $x_{0}\in \left\{x_{0}(t)-4,x_{0}(t)-3,\ldots ,x_{0}(t)+3,x_{0}(t)+4\right\}$, $y_{0}\in \left\{y_{0}(t)-4,y_{0}(t)-3,\ldots ,y_{0}(t)+3,y_{0}(t)+4\right\}$, and r∈{r(t)−4,r(t)−3,…,r(t)+3,r(t)+4}. Other processes in this step were similar to those of the rough pupil detection step, in terms of estimating the pupil center ($x_{0},y_{0}$) and radius r for each frame t. The method described in Section 4.2.1 was used to re-estimate the parameter with an accuracy of 1 pixel. The result of the estimation was defined by $x_{0}(t)$, $y_{0}(t)$, and r(t).

Finally, in the subpixel detection step, the search range was further limited, using the parameters that were estimated in the precise detection step. The method described in Section 4.2.1 was used again, in order to re-estimate the parameters with an accuracy of 1/4 pixels. The search ranges for the pupil center $\left(x_{0},y_{0}\right)$ and radius r were limited to $x_{0}\in \left\{x_{0}(t)-1,x_{0}(t)-0.75,\ldots ,x_{0}(t)+0.75,x_{0}(t)+1\right\}$, $y_{0}\in \left\{y_{0}(t)-1,y_{0}(t)-0.75,\ldots ,y_{0}(t)+0.75,y_{0}(t)+1\right\}$, and r∈{r(t)−1,r(t)−0.75,…,r(t)+0.75,r(t)+1}.

### 4.3. Estimation of the Optimal Flatness Parameter q

**Figure 5:**
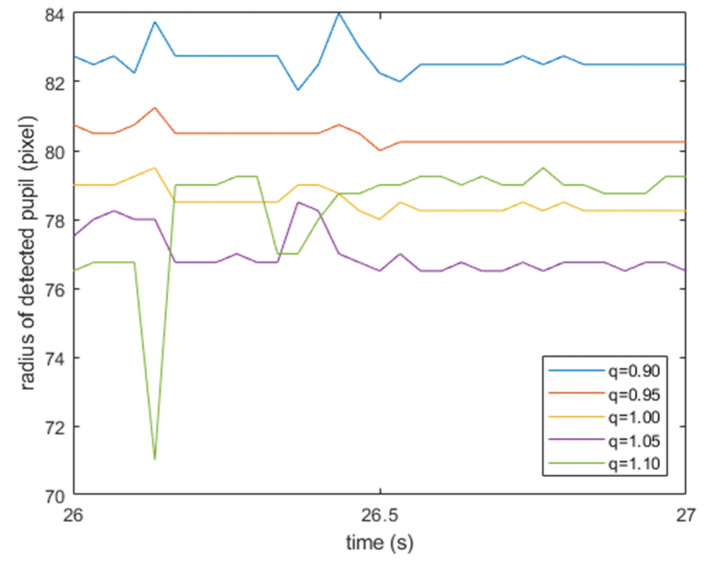
Sample of r(t) for varying values of q.

According to the proposed method described in Section 4.2, the waveforms of the center coordinates $x_{0}(t)$, $y_{0}(t)$ and radius r(t) of the pupil were estimated for each flatness parameter q∈{0.90,0.95,1.00,1.05,1.10}. The magnitude of the fluctuation of the radius r(t) can be used as a measure of estimation accuracy—that is, the best selection for the flatness parameter—as the radius r(t) does not change much, even if the center coordinates $x_{0}(t)$, $y_{0}(t)$ vary with nystagmus. Therefore, the optimum flatness parameter q is defined as the value that minimizes the magnitude of fluctuation of the radius r(t). Figure 5 shows examples of the radius r(t) estimated with each q∈{0.90,0.95,1.00,1.05,1.10} for the same eye video. It can be concluded that q=0.95 was optimal, as the radius r(t) had minimum fluctuation. The specific calculation method for the magnitude of fluctuation is summarized in Appendix B.

## 5. Results

**Figure 6:**
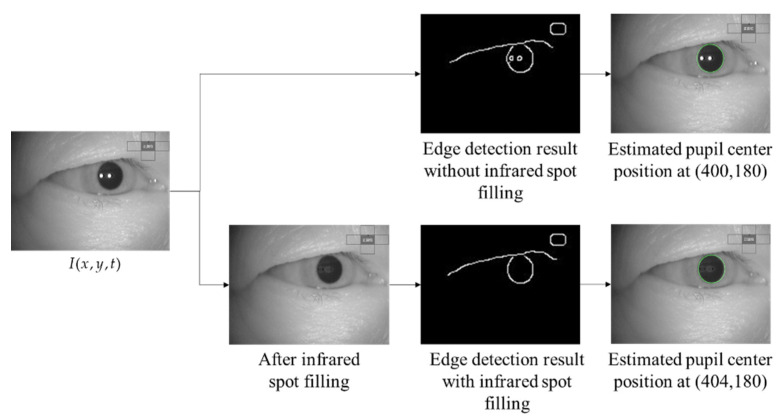
Sample of infrared spot filling to the detection result.

The existence of infrared spots influences the edge detection process for detecting the pupil contour, based on the intensity gradient between the pupil and the iris. Removing the spots is essential, as they decrease the accuracy of pupil detection. Due to the spots in the eye image, the edge detection step will also discern another circular border inside the pupil area. Consequently, when calculating the degree of similarity between the ellipse pattern and the edge image, the circular border from the spots shifts the pupil’s estimated center. Figure 6 shows a comparison of edge detection results with and without the infrared spot filling process.

**Figure 7:**
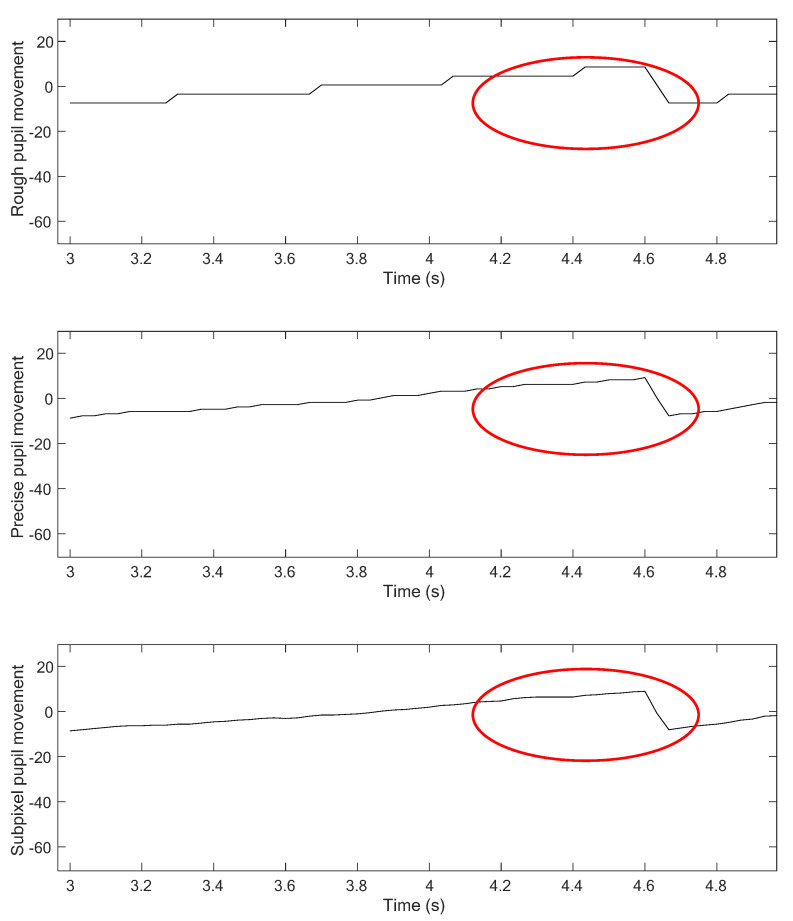
Comparison of rough, precise, and subpixel detection results.

**Figure 8:**
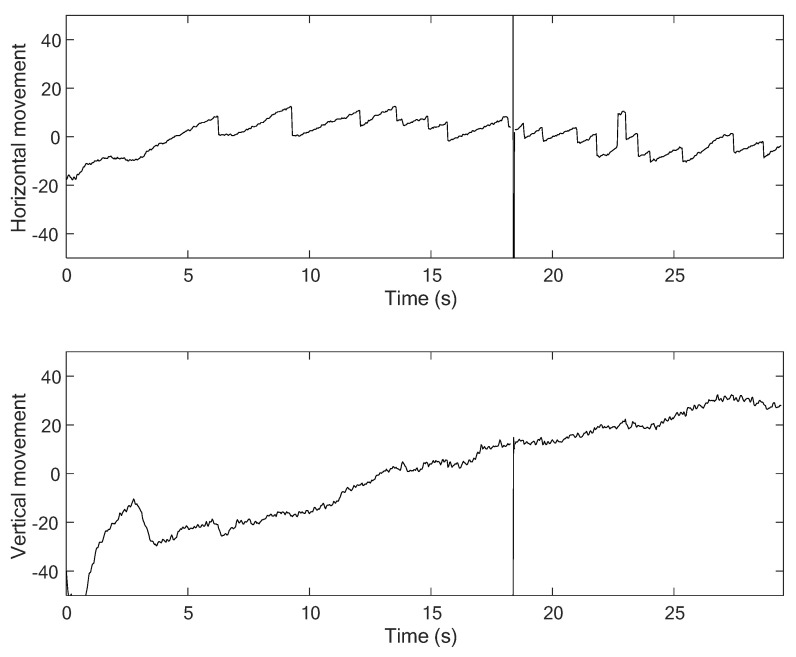
Sample of the nystagmus waveform generated by the proposed method.

Figure 7 shows comparison results from the three-step precision improvement process described in Section 4.2.2. It can be observed that the nystagmus waveform becomes smoother at each step, due to the improvement of the pixel-order estimation. The pixel-order estimation is improved from 4 pixels to 1 pixel, and then to 1/4 pixel, as highlighted by the red ellipse. Figure 8 shows a sample of a nystagmus waveform generated by the proposed method. The waveform represents the pupil center position, based on its horizontal and vertical movement.

## 6. Evaluation

### 6.1. Performance Evaluation for Partially Shown Pupil

**Figure 9:**
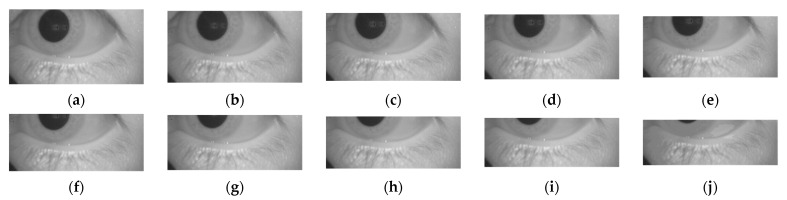
Illustration of pupil cropping: (**a**) 100%; (**b**) 90%; (**c**) 80%; (**d**) 70%; (**e**) 60%; (**f**) 50%; (**g**) 40%; (**h**) 30%; (**i**) 20%; and (**j**) 10%.

As was highlighted in Section 1, patients who complain of dizziness often have difficulties in keeping their eyes open, which may require nystagmus to be measured from a semi-open state. Therefore, the performance of the proposed method was evaluated for eye movement videos under the condition that the video only shows a partial part of the pupil. Therefore, the video was cropped to show 100% to 10% of the pupil, with a gradual decrement by 10%. In this research, the removal of the pupil part started from the top area of the pupil. Figure 9 shows an illustration of pupil cropping.

We calculated the Mean Square Error (MSE) between pupil position from a cropped pupil and fully visible pupil to assess the accuracy of the method. Based on visual observations, the obtained pupil center results for both methods had some outlier detections. In order to consider the outliers, outlier detection was not be included in the MSE calculation if the difference in pupil center position was equal to or larger than 20 pixels.

The MSE for all video frames was calculated as

(7) $$MSE=\frac{1}{T}\sum_{t=1}^{T}\left({\left(\frac{x_{0}(t)-x_{0}{}^{\prime }(t)}{N_{x}}\right)}^{2}+{\left(\frac{y_{0}(t)-y_{0}{}^{\prime }(t)}{N_{y}}\right)}^{2}\right),$$

where ($x_{0}(t),y_{0}(t)$) and ($x_{0}{}^{\prime }(t),y_{0}{}^{\prime }(t)$) are the pupil center positions in the videos with whole pupils and partial pupils, respectively.

**Figure 10:**
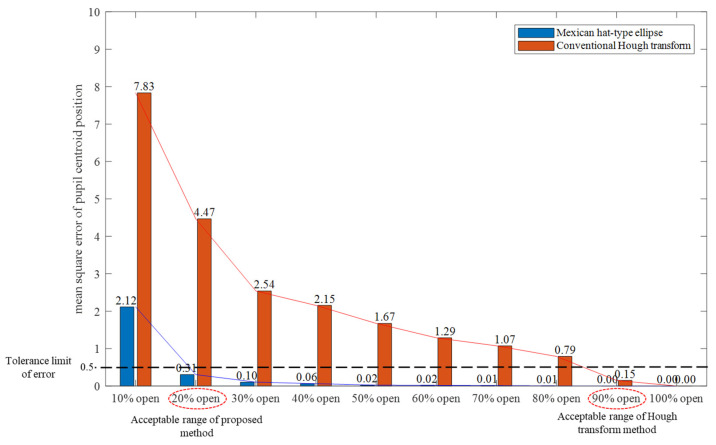
Comparison of MSE calculation results from the proposed Mexican hat-type ellipse pattern matching and the conventional Hough transform method.

We evaluated the performance of the proposed method in comparison to that of the conventional Hough transform method. For the evaluation, the MSE of each video is averaged, in order to obtain the mean MSE for each percentage of the visible pupil.

(8) $$\bar{MSE}=\frac{1}{V}\sum_{v=1}^{V}MSE(v),$$

where MSE(v) is the MSE from video number v∈{1,2,…,V}, where V defines the total number of videos. Figure 10 shows the comparison results as a bar graph. In general, the Mexican hat-type ellipse pattern matching achieved a lower MSE, compared to the conventional Hough transform method. Specifically, if we define the acceptable range of error limit tolerance as 0.5 MSE, the performance of the proposed method achieved MSE values below the 0.5 limit until 20% of the pupil was visible. In other words, the proposed method can detect and track the movement of the center of the pupil almost as accurately as when 100% of the pupil is visible. In comparison, the conventional Hough transform method indicated a low MSE value under the 0.5 limit if only 90% of the pupil was visible. If the pupil was occluded more than 20%, the MSE value of the conventional Hough transform method increased significantly.

**Figure 11:**
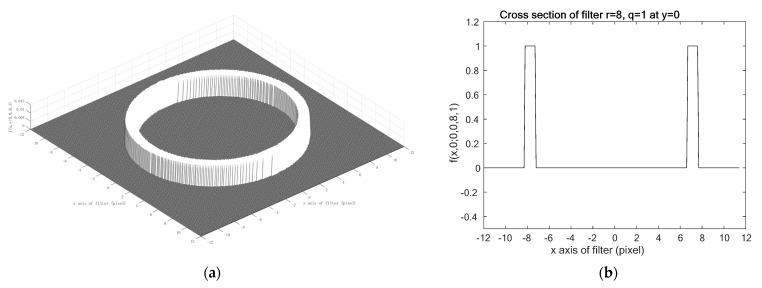
The example of the conventional Hough transform pattern with a uniform-valued ring: (**a**) Bird’s-eye view; and (**b**) cross-section at y = 0.

The reason why the proposed method achieved higher estimation accuracy than the conventional Hough transform is described as follows. In the conventional Hough transform, the pixels within a certain width range are aggregated with equal weight for the target shape. Then, the maximum aggregate is used to estimate the parameters of the target shape. Therefore, circle detection by the conventional Hough transform is equivalent to pattern matching using a pattern with a uniform weight pattern, as shown in Figure 11. However, if the target shape has a blurry boundary that is not always clear, such as a whole pupil, the maximum degree of similarity $h(x_{0},y_{0},r,q;t)$ cannot be achieved, thus deteriorating the estimation accuracy. Therefore, we calculated the similarity degree $h(x_{0},y_{0},r,q;t)$ using the Mexican hat-type ellipse pattern, as shown in Figure 4a. The proposed method generates a sharp peak for boundary detection. Thus, it is expected to improve estimation accuracy.

**Figure 12:**
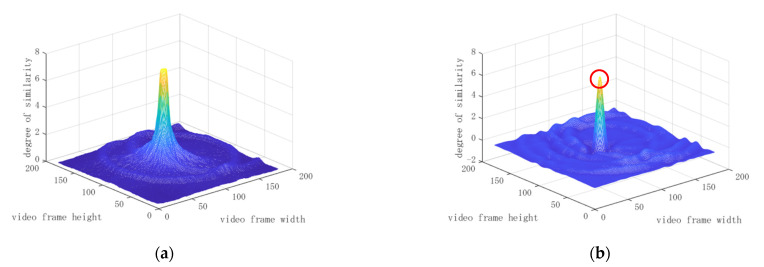
The difference in peak sharpness for the evaluation function $h(x_{0},y_{0},r,q;t)$: (**a**) Conventional Hough transform; and (**b**) Mexican hat-type ellipse pattern.

Figure 12 shows the comparison result of the evaluation function $h(x_{0},y_{0},r,q;t)$ for the conventional Hough transform and the Mexican hat-type ellipse pattern. Figure 12a shows that the conventional Hough transform resulted in a flat peak, with some peaks resulting in the same degree of similarity. Therefore, it could not lead to a single maximum value of $h(x_{0},y_{0},r,q;t)$, representing the pupil center position. Meanwhile, the proposed Mexican hat-type pattern resulted in a single maximum peak value. Figure 12b shows the maximum peak, highlighted as a red circle, as the candidate for the pupil center.

As the performance of the proposed method is reliant on the detected pupil’s shape, any artifacts that distort the pupil shape, such as accidents and optical diseases, will influence the results. For example, pupil abnormalities caused by Colobomas, Adie syndrome, or severe Uveitis can influence the accuracy of pupil tracking. Cloudiness in the cornea, such as Glaucoma and Cataracts, will also influence the accuracy of pupil tracking. Recommendations for further research include Nystagmus estimation for this abnormal and distorted pupil shape.

### 6.2. Performance Evaluation Using the Labelled Pupil in the Wild Data Set

Using Equations (7) and (8), pupil center information from the proposed method was compared with the ground truth of the LPW data set. Using a similar approach, the performance of the conventional Hough transform method was also calculated. The proposed method achieved an MSE of 1.47, while the conventional Hough transform method achieved an MSE of 9.53.

### 6.3. Medical Specialist Validation

In this research, the Mexican hat-type ellipse pattern matching for detecting the pupil center was also evaluated using an expert validation approach. The expert validation approach was conducted by asking three medical specialists to evaluate the nystagmus waveform obtained from the proposed method. Then, the medical specialists wrote their reviews, regarding what the waveform represented. The medical specialist also commented on the eye movement video conditions and mentioned challenges in diagnosing the nystagmus state of disease.

Based on the medical specialists’ reviews, the nystagmus waveform from the proposed method was evaluated clinically. The waveform could be used to assess unstable nystagmus without any problem. The proposed method can also detect the correct direction of the nystagmus case, and the detection was also accurate for both rapid and slow phases of nystagmus.

**Figure 13:**
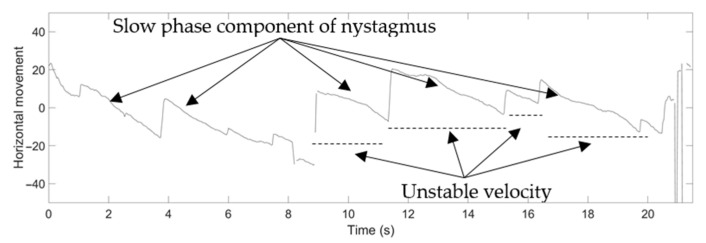
Nystagmus waveform from the proposed method for Video No. 1, horizontal movement of the pupil.

**Figure 14:**
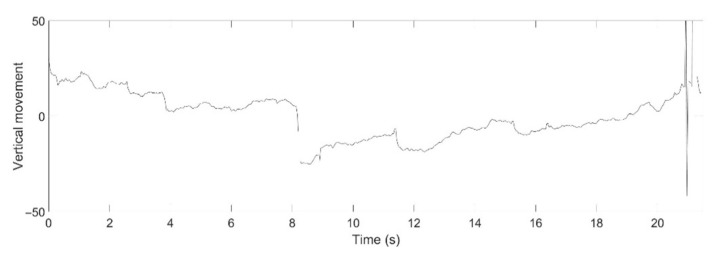
Nystagmus waveform from the proposed method for Video No. 1, vertical movement of the pupil.

For example, the medical specialists highlighted the slow phase component of nystagmus in the horizontal direction of Video No. 1. This slow phase component is shown in Figure 13 as a nystagmus waveform generated by the proposed method. The medical specialist noticed that even the velocity of the slow phase was unstable; however, the system can be used to evaluate the nystagmus. In addition, as vertical nystagmus was not observed in the video, the slow phase was also undetected in the pupil vertical movement waveform, as shown in Figure 14.

**Figure 15:**
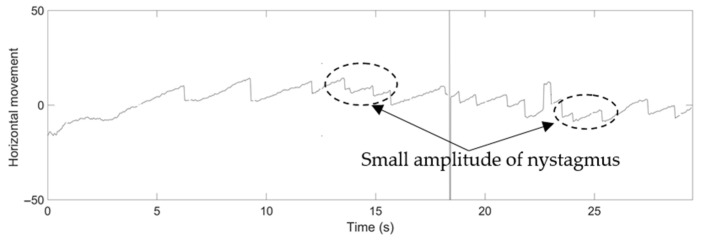
Nystagmus waveform from the proposed method for Video No. 28, horizontal movement of the pupil.

In the case of nystagmus with high frequency, the proposed method could accurately capture the nystagmus. Furthermore, in the case of a low frequency of nystagmus, which is difficult to evaluate with the naked eye, it could be confirmed and detected in the waveform. An example of this can be seen in the nystagmus waveform for Video No. 28, as shown in Figure 15. The small amplitude of nystagmus was captured well by the proposed method for rapid and slow phase components in horizontal pupil movement.

**Figure 16:**
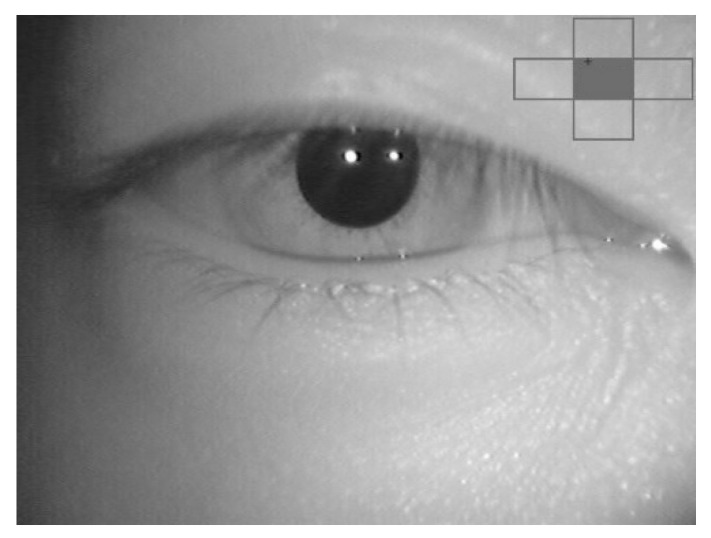
Sample of a video frame from Video No. 2.

**Figure 17:**
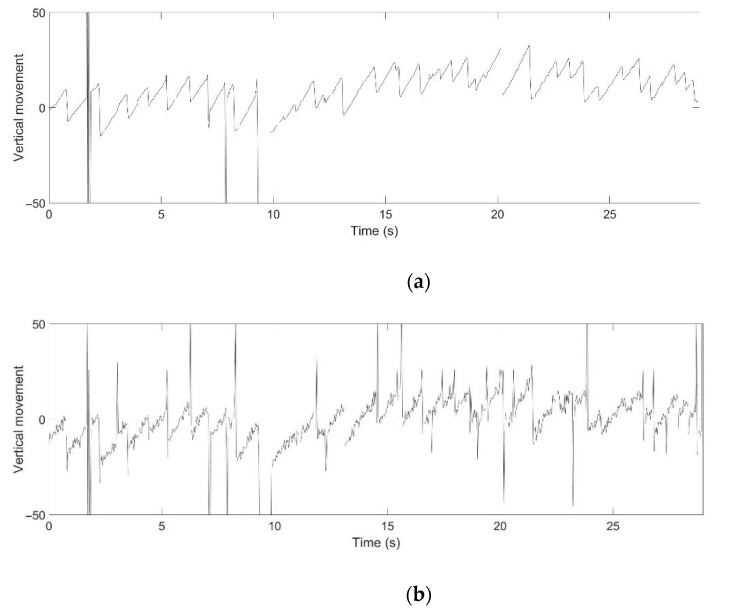
Nystagmus waveform for Video No. 2: (**a**) Using the proposed method and; (**b**) using the conventional Hough transform method.

While the performance of the proposed method was well-recognized with a wide eyelid gap, the medical specialist also agreed that the waveform can be used to confirm nystagmus when the eyelid gap is narrow. The medical specialist mentioned that the condition of the narrow eyelid gap is difficult to evaluate. The entire iris is not visible in some videos, as some patients had difficulty in fully opening their eyes. However, the waveform can track pupil movement in both horizontal and vertical directions. The medical specialist mentioned that the waveform could still be used when up to 30% of the pupil was shown. For example, the medical specialist mentioned that the patient had difficulty opening her eyes in Video No. 2. Figure 16 shows a video frame from Video No. 2, which represents this condition. Figure 17a shows the nystagmus waveform that was obtained from Video No. 2. Based on this waveform, the vertical component of the nystagmus was well-captured by the proposed method. In comparison, Figure 17b shows the nystagmus waveform from the conventional Hough transform method. The waveform had a high vibration of the vertical component of the nystagmus, due to the problem illustrated in Figure 12.

**Figure 18:**
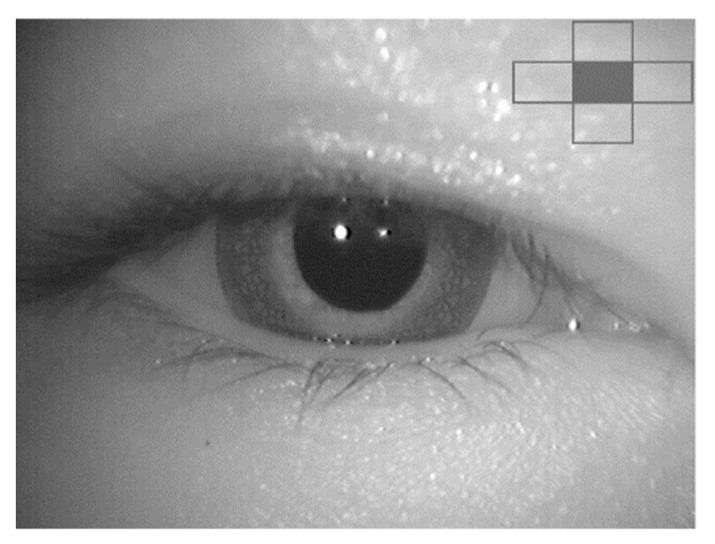
Sample of a video frame from Video No. 11.

**Figure 19:**
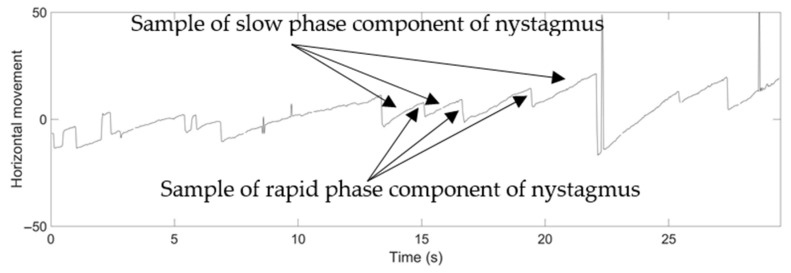
Nystagmus waveform from the proposed method for Video No. 11, horizontal movement of the pupil.

In addition, the presence of contact lenses in the video does not affect the performance of the proposed method. Figure 18 shows a sample of a video frame from Video No. 11 which represents this condition, while Figure 19 shows a waveform that captures the horizontal rapid and slow phases of nystagmus for Video No. 11.

The medical specialist also recommended improving the infrared camera’s specifications, as there was a limit, in terms of capture capacity, which prevented accurate evaluation of the rapid phase of nystagmus. The medical specialist also mentioned that the rotational component of nystagmus should be included in the waveform. Details of the medical specialists’ review are provided in Appendix A, Table A2.

## 7. Conclusions

The principal purpose of this research was successfully achieved. Mexican hat-type ellipse pattern matching for detecting the center of a partially open pupil was proposed. Experiments using the implemented method on 37 eye videos were evaluated. The Mexican hat-type ellipse pattern matching approach achieved better performance, compared to the conventional Hough transform method. The evaluation also showed the robust performance of the proposed method, even when only 20% of the pupil was shown. Further evaluation of the performance of the proposed method using the LPW data set also showed that it can achieve a lower MSE, compared to the conventional Hough transform method. A review by medical specialists also provided evidence that the proposed method can support their diagnosis in the case of a low frequency of nystagmus, which is difficult to evaluate with the naked eye. In addition, the waveform generated by the proposed method can reproduce eye movement in horizontal and vertical directions under the conditions of a narrow eyelid gap, which is difficult to evaluate. Therefore, the contributions of this research could lead to reasoning and diagnostic improvement of medical specialists, in the case of nystagmus estimation for dizziness diagnosis.

## Data Availability

Data sharing for videos listed in Table A1 are not applicable to this article. LPW data can be accessed at www.mpi-inf.mpg.de/departments/computer-vision-and-machine-learning/research/gaze-based-human-computer-interaction/labelled-pupils-in-the-wild-lpw (accessed on 26 January 2021).

## Appendix A

**Table A1:** Summary of subject videos.

Video No.	State of Disease	Gender	Age	Total Duration (s)
1	Meniere’s disease	Female	44	21
2	Meniere’s disease	Female	60	28
3	Meniere’s disease	Male	65	23
4	Meniere’s disease	Male	81	29
5	Benign Paroxysmal Positional Vertigo	Female	80	18
6	Benign Paroxysmal Positional Vertigo	Female	75	29
7	Benign Paroxysmal Positional Vertigo	Female	80	29
8	Benign Paroxysmal Positional Vertigo	Male	73	29
9	Benign Paroxysmal Positional Vertigo	Female	80	29
10	Benign Paroxysmal Positional Vertigo	Male	73	29
11	Right vestibular disorder	Female	31	29
12	Right semicircular canal paralysis	Female	54	17
13	Meniere’s disease	Male	37	19
14	Meniere’s disease	Male	37	18
15	Left vestibular neuritis	Male	59	29
16	Left vestibular disorder	Male	55	27
17	Right sudden deafness	Male	47	29
18	Left Anterior Inferior Cerebellar Artery	Female	64	29
19	Right hunt	Female	54	19
20	Left vascular nerve compression	Female	68	18
21	Right hunt	Female	70	20
22	Right vestibular neuritis	Male	67	12
23	Right Meniere	Male	67	26
24	Right hunt	Male	80	21
25	Left vestibular neuritis	Male	71	11
26	Meniere’s disease	Female	47	29
27	Meniere’s disease	Female	72	29
28	Meniere’s disease	Male	48	29
29	Meniere’s disease	Male	57	8
30	Meniere’s disease	Male	36	29
31	Meniere’s disease	Male	65	29
32	Medulla oblongata bleeding	Male	44	13
33	Cerebellar disease Nystagmus for lower eyelid	Female	41	14
34	Neurovascular compression syndrome	Male	37	15
35	Spinocerebellar degeneration	Male	65	17
36	Congenital nystagmus	Male	28	7
37	Multiple system atrophy	Male	57	18

**Table A2:** Summary of medical specialist review.

Video No.	Review from Medical Specialist A, B, and C
1	A. This video is the nystagmus of a patient in the intermittent phase of Meniere’s disease. The patient can open her eyes sufficiently. Therefore, the eyelid gap is wide, and the iris is well captured. The system captures the horizontal slow-phase component of nystagmus. B. This video is a nystagmus finding in the interictal phase. The patient was able to open her eyes, and almost all of the iris is shown. A rapid eye movement in the right horizontal direction and a slow phase are recorded in the measurement waveform reproducing the actual nystagmus findings. Vertical nystagmus was not observed in the video, and the slow phase undetected in the measurement waveform. Therefore, the measurement waveform can reproduce the actual nystagmus. C. The system captures the horizontal nystagmus. Although the two slow phase velocities in the nystagmus are unstable, the system can be evaluated as generally measuring them without problems.
2	A.The video shows the nystagmus of a patient in the acute phase of Meniere’s disease. The patient is in the acute phase of a vertiginous attack and may have difficulty opening her eyes sufficiently. As a result, the eyelid gap is narrow, and it is usually difficult to capture the iris. However, the system captures a slow phase component of the nystagmus in the horizontal and vertical directions. B. The video shows the nystagmus of a patient in the acute phase of Meniere’s disease. The patient seems to have difficulty opening her eyes sufficiently. As a result, the eyelid gap is relatively narrow, and it is usually difficult to capture the iris completely. However, the waveform of this system captures the horizontal and vertical slow phase components of nystagmus. Therefore, this system can be used even when the eyelid gap is narrow. C. The vertical component of the nystagmus is well captured in the system. On the other hand, the horizontal part was lacking, resulting in some confusion in the results. This case is also in the acute stage of vertigo, and the nystagmus components may include various directions. Therefore, further analysis of the rotation component may help us to detect the disease more clearly.
4	A. The video shows the nystagmus of a patient in the acute phase of Meniere’s disease. The patient is in the acute phase of a vertiginous attack and may have difficulty opening his eyes sufficiently. As a result, the eyelid gap is narrow, and it is usually difficult to capture the iris. However, the system can capture horizontal and vertical slow phase components of nystagmus during head-turning. B. The video shows the nystagmus of left Meniere’s disease in the paroxysmal period. The entire iris is well captured in the image. The nystagmus is mainly in the right horizontal direction and has a slight rotation component in the image. The rapid phase and slow phase components of the right direction are evaluated in the measurement waveform. Vertical eye movements were not shown in any waveforms suggestive of nystagmus. C. Although the eye movements could not be captured in the second half, the horizontal component was accurately captured in the first half. This is a case where the goggles used for recording need to be improved, and this analysis software is commendable.
9	A. The video shows the nystagmus of a patient with benign paroxysmal positional vertigo. The patient is in the acute phase of a vertiginous attack and may have difficulty opening her eyes sufficiently. As a result, the eyelid gap is narrow, and it is usually difficult to capture the iris. However, this system captures the horizontal slow phase component of nystagmus. B. The video shows head-on nystagmus of BPPV. The nystagmus is mainly in the right horizontal direction and a slight rotation component in the image. The entire iris is unobserved in many cases, and the iris is unobserved in the eyelid gap in about 1/3 of the video. However, rapid and slow phase components in the right direction are evaluated in the measurement waveform. The rapid phase, which is presumably downward due to the gyration component, is reproduced in the vertical direction. C. Although the frequency of nystagmus resolution is high in this case, the nystagmus is accurately captured in the horizontal component. Although some of the rapid phases are not fully grasped, the waveform can be evaluated as nystagmus in patients with vertigo, especially BPPV.
11	A. The video shows the nystagmus of a patient with a vestibular disorder. The patient has difficulty opening her eyes sufficiently. As a result, the eyelid gap is narrow, and it is usually difficult to capture the iris. However, this system captures the horizontal slow-phase component of nystagmus. B. Leftward nystagmus on the healthy side due to right vestibular dysfunction was observed. Although contact lenses were worn by the patient, most of the iris was visible. The left horizontal rapid-phase and slow-phase components are evaluated in this measurement waveform. The presence of contact lenses does not affect the analysis. C. Although nystagmus is difficult to evaluate with the naked eye due to its low frequency, this analysis confirms a horizontal component. The absence of a vertical element makes it possible to evaluate nystagmus as an HC-BPPV.
14	A. The video shows the nystagmus of a patient with Meniere’s disease in the intermittent phase. The patient has difficulty opening his eyes sufficiently. As a result, the eyelid gap is narrow, and it is usually difficult to capture the iris. However, this system captures the horizontal slow-phase component of nystagmus. B. The eye movements are mainly left horizontal nystagmus, but there is a left downward oblique movement once every few strikes. In the measurement waveform, the slow phase in the left horizontal direction is visible. The vertical analysis also shows the waveform suggesting downward eye movement due to obliquity. Subtle vertical eye movements were captured. C. The nystagmus component in Meniere’s disease is often complex. Even in cases such as the present case, where the nystagmus appears to have only a horizontal component to the naked eye, the analysis suggests that a vertical component is also present. The results of this analysis are sufficient for the analysis of nystagmus and may have clinical application.
28	A. The video shows the nystagmus of a patient with Meniere’s disease in the intermittent phase. The patient can open his eyes sufficiently. Therefore, the eyelid gap is wide, and the iris is well captured. The system captures the horizontal slow-phase component of nystagmus. B. The patient with Meniere’s disease was in the interictal phase. The patient was able to maintain normal eye-opening. The left horizontal nystagmus is observed in the video. The left rapid-phase and slow-phase components in the horizontal movement are evaluated in the measurement waveform. This system captures nystagmus with low frequency in the intermittent phase. C. The video shows an example of what might be mistaken for impulsive eye movements by the naked eye because of the small amplitude of nystagmus. However, the analysis captures horizontal nystagmus. The fact that the absence of a vertical component can be confirmed is also commendable.
31	A. The video shows the nystagmus of a patient with Meniere’s disease in the intermittent phase. The patient can open his eyes sufficiently. Therefore, the eyelid gap is wide, and the iris is well captured. The system captures horizontal and vertical slow-phase components of nystagmus. B. The amplitude of the nystagmus is low, and the blink frequency is high even with the eye movement images because the patient with Meniere’s disease is in the intermittent phase. Therefore, it is not easy to grasp eye movements. Nevertheless, the measurement waveform shows the rapid and slow phase components in the left horizontal direction. On the other hand, the vertical measurement shows nystagmus-like waveforms with rapid-phase and slow-phase components in the upper eyelid direction. However, it is difficult to identify them in the actual eye movement images. C. It is difficult to differentiate between peripheral and central nystagmus at first glance, as this case has both large and small amplitude components. The patient also had a brain tumor, and the presence of vertical nystagmus may provide clinically useful information, which is commendable.
32	A. The video shows the nystagmus of a patient with a Medulla oblongata bleeding. The patient is in the acute phase of a vertiginous attack and may have difficulty opening his eyes sufficiently. As a result, the eyelid gap is narrow, and it is usually difficult to capture the iris. However, this system captures horizontal and vertical slow-phase components of nystagmus. B. The eye movement images show that the eye is displaced to the right and that it is difficult to capture the entire iris due to the narrow eyelid gap. Nevertheless, leftward nystagmus observed frequently can be seen. Although it lacks continuity in some places, the measurement waveform shows a rapid phase and a slow phase in the left horizontal direction. C. Although the frequency and amplitude of the nystagmus were considerable, the rapid phase of the horizontal component was not captured, which shows that the accuracy of the evaluation of the rapid phase is limited. However, it is sufficient to evaluate the slow phase. The fact that the vertical component is also captured is commendable. The fact that the vertical component also does not capture the rapid phase seems to be due to the limitation of the capturing capability of the infrared camera. Therefore, it is desirable to use a more powerful camera to capture the rapid phase more clearly.
33	A. The video shows the nystagmus of a patient with cerebellar disease nystagmus for the lower eyelid. The patient can open her eyes sufficiently. Therefore, the eyelid gap is wide, and the iris is well captured. The system captures the vertical slow-phase components of nystagmus. B. The entire iris is captured in the second half of the recording, and rhythmic downward eye movement can be confirmed in the eye movement images. In the measurement waveform, the rapid downward and slow phase is evaluated in the second half of the images. There is a scene where the eyeball is significantly displaced to the right in the first half of the images. In such a situation in which the iris is partially missing, the measurement waveform does not reproduce the nystagmus. C. The patient came to our hospital with a complaint of balance disorder due to spinocerebellar degeneration. The downward nystagmus was accurately captured, and the presence of a weak horizontal component could be confirmed. The fact that nystagmus can be recognized even when the eyelid is lowered and half of the iris cannot be captured is commendable.
35	A. The video shows the nystagmus of a patient with Spinocerebellar degeneration. The patient has difficulty opening his eyes sufficiently. As a result, the eyelid gap is narrow, and it is usually difficult to capture the iris. However, this system captures the vertical slow-phase component of nystagmus. B. Although about 1/3 of the iris is blocked by the upper eyelid in the eye movement images, the downward nystagmus can be recognized. Some oblique eye movements are included in the images. The waveform shows a rapid phase and a slow phase in the vertically downward direction. The waveform captures the nystagmus even if the entire iris is not recorded. A rightward movement due to the actual oblique movement is observed in the horizontal analysis. However, the rightward movement cannot be evaluated as a clear rapid-slow phase. C. The patient came to our hospital with a complaint of balance disorder due to spinocerebellar degeneration. The downward nystagmus was accurately captured, and the presence of a weak horizontal component could be confirmed. The fact that the nystagmus can be recognized even when the eyelid is lowered and half of the iris cannot be captured, is commendable.
37	A. The video shows the nystagmus of a patient with multiple system atrophy. The patient has difficulty opening his eyes sufficiently. As a result, the eyelid gap is narrow, and it is usually difficult to capture the iris. However, this system captures horizontal and vertical slow-phase components of nystagmus. B. The nystagmus is predominantly downward and oblique with a suitable horizontal component in the eye movement images. Although the entire iris was not visible in some areas, the measurement waveform reproduced both horizontal and vertical eye movements. C. The video shows a case of multiple system atrophy and central vertigo. Vertical nystagmus is the predominant finding. It can be seen that there is also a horizontal component. Clinically, the results of the analysis are consistent.

## Appendix B

The fluctuation of r(t) is calculated as follows: First, the absolute difference between r(t) and r(t+1) is calculated, using

(A1) rdiff(t)=|r(t)−r(t+1)|,

where rdiff(t) is the absolute difference. Then, rdiff(t) is categorized into false detection and true detection categories. The category is divided based on the value of rdiff(t), which represents the variation of r(t). If rdiff(t) is larger than 10 pixels, then the detection is categorized as false detection. Otherwise, rdiff(t) is categorized as true detection.

The average of false detection occurrence is defined as $rdiff_{false}$, which is calculated using:

(A2) $$rdiff_{false}=\frac{1}{T}\sum_{t=1}^{T}occ_{false}(t),$$

in which,

(A3) $$occ_{false}(t)=\{\begin{array}{c} 10,rdiff(t)>10\\ 0 \end{array},$$

where $occ_{false}(t)$ is the false detection occurrence.

The true detection average is calculated based on the total occurrence of rdiff(t) which is lower than 10 pixels. The total occurrence of true detections, defined as $countrdiff_{true}$, is calculated using:

(A4) $$countrdiff_{true}=\sum_{t=1}^{T}occ_{true}(t),$$

where

(A5) $$occ_{true}(t)=\{\begin{array}{c} 1,rdiff(t)\leq 10\\ 0 \end{array},$$

in which $occ_{true}(t)$ is a variable that takes a binary value, representing the occurrence of a true detection by 1; otherwise, it is 0. Then, the total value of rdiff(t) for these true detections is defined as $totaldiff_{true}$, which is also calculated using:

(A6) $$totaldiff_{true}=\sum_{t=1}^{T}value_{true}(t),$$

in which,

(A7) $$value_{true}(t)=\{\begin{array}{c} rdiff(t),rdiff(t)\leq 10\\ 0 \end{array},$$

where $value_{true}(t)$ is a variable that summarizes the value of true detection from rdiff(t). Finally, $rdiff_{true}$, which defines the true detection average, is calculated using

(A8) $$rdiff_{true}=\frac{totaldiff_{true}}{countdiff_{true}}.$$

Based on Equations (2) and (8), the fluctuation of r(t) is calculated based on $rdiff_{false}$ and $rdiff_{true}$, using:

(A9) $$rdiff_{all}=rdiff_{true}+rdiff_{false},$$

where $rdiff_{all}$ is the fluctuation of r(t). As previously mentioned at the beginning of this section, the optimum q is defined as that which results in the minimum $rdiff_{all}$. Finally, all of the $x_{0}(t)$, $y_{0}(t)$, and r(t) values from the optimum q are collected as the final detection result.
